# 

**Published:** 1882-06

**Authors:** 


TWENTY-NINTH YEAR.

### HALL'S JOURNAL OF HEALTH

*E. H. GIBBS, A.M., M.D., Editor,*

No. 135 EIGHTH STREET (Near Broadway), NEW YORK.

TERMS: $2 a Year, or $1 for Six Months. Single number, 20 cts.

Entered as Second class Matter in the Post-Office in New York.

